# *Faecalibacterium prausnitzii* Strain HTF-F and Its Extracellular Polymeric Matrix Attenuate Clinical Parameters in DSS-Induced Colitis

**DOI:** 10.1371/journal.pone.0123013

**Published:** 2015-04-24

**Authors:** Oriana Rossi, M. Tanweer Khan, Martin Schwarzer, Tomas Hudcovic, Dagmar Srutkova, Sylvia H. Duncan, Ellen H. Stolte, Hana Kozakova, Harry J. Flint, Janneke N. Samsom, Hermie J. M. Harmsen, Jerry M. Wells

**Affiliations:** 1 Host-Microbe Interactomics Group, Animal Science Department, University of Wageningen, Wageningen, The Netherlands; 2 Department of Cell Biology, section Membrane Cell Biology, University Medical Center Groningen, Groningen, The Netherlands; 3 Institute of Microbiology of the Academy of Sciences of the Czech Republic, Prague, Czech Republic; 4 Microbial Ecology Group, Rowett Institute of Nutrition and Health, University of Aberdeen, Aberdeen, United Kingdom; 5 Department of Pediatrics, Division of Gastroenterology and Nutrition, Erasmus Medical Center—Sophia Children's Hospital, Rotterdam, The Netherlands; University of Ulm, GERMANY

## Abstract

A decrease in the abundance and biodiversity of intestinal bacteria within the Firmicutes phylum has been associated with inflammatory bowel disease (IBD). In particular, the anti-inflammatory bacterium *Faecalibacterium prausnitzii*, member of the Firmicutes phylum and one of the most abundant species in healthy human colon, is underrepresented in the microbiota of IBD patients. The aim of this study was to investigate the immunomodulatory properties of *F*. *prausnitzii* strain A2-165, the biofilm forming strain HTF-F and the extracellular polymeric matrix (EPM) isolated from strain HTF-F. For this purpose, the immunomodulatory properties of the *F*. *prausnitzii* strains and the EPM were studied *in vitro* using human monocyte-derived dendritic cells. Then, the capacity of the *F*. *prausnitzii* strains and the EPM of HTF-F to suppress inflammation was assessed *in vivo* in the mouse dextran sodium sulphate (DSS) colitis model. The *F*. *prausnitzii* strains and the EPM had anti-inflammatory effects on the clinical parameters measured in the DSS model but with different efficacy. The immunomodulatory effects of the EPM were mediated through the TLR2-dependent modulation of IL-12 and IL-10 cytokine production in antigen presenting cells, suggesting that it contributes to the anti-inflammatory potency of *F*. *prausnitzii* HTF-F. The results show that *F*. *prausnitzii* HTF-F and its EPM may have a therapeutic use in IBD.

## Introduction

Ulcerative colitis (UC) and Crohn’s disease (CD), two forms of inflammatory bowel disease (IBD), are driven by an aberrant inflammatory T cell response to intestinal microbiota in a genetically susceptible host. Profound changes in the diversity and composition of the microbiota are associated with UC and CD [1,2]. A decrease in the frequency of the phyla Bacteroidetes and Firmicutes and an increase of Proteobacteria and Actinobacteria have been observed in the faecal microbiota of IBD patients [3]. In particular, *Faecalibacterium prausnitzii*, a member of the Firmicutes phylum and one of the most abundant species in the healthy human colon [4], is underrepresented in the microbiota of IBD patients [5]. Mucosal-associated counts of *F*. *prausnitzii* from ileal biopsies are also lower in CD patients with active disease than in patients in remission [6,7]. *F*. *prausnitzii* was reported to be an anti-inflammatory bacterium on account of its capacity to induce high amounts of IL-10 in human peripheral blood mononuclear cells (PBMCs, [7]). Treatment of Caco2 cells with *F*. *prausnitzii* culture supernatant was reported to reduce IL-1β-induced NF-κB activation and secretion of IL-8. This was attributed to an as yet unidentified factor secreted in the culture medium as it was not observed using 40 mM butyrate, UV-Killed bacteria or bacterial DNA, membranes and cytoplasmic extract [7]. Administration of *F*. *prausnitzii* strain A2-165 and its culture supernatant have been shown to protect against 2,4,6-trinitrobenzenesulfonic acid (TNBS)-induced colitis in mice [7]. This model is thought to resemble CD because the resulting mucosal inflammation is mediated by a T helper 1 (Th1) response with excessive production of IFN-γ, TNF-α and IL-12. Additionally, intragastric administration of *F*. *prausnitzii* A2-165 and its culture supernatant have a protective effect against dinitrobenzenesulfonic (DNBS)-induced chronic colitis [6].

The aim of this study was to test the capacity of two different *F*. *prausnitzii* strains to suppress inflammation in the mouse dextran sodium sulphate (DSS) colitis model. The strains tested were A2-165 and the newly characterized HTF-F. Here we showed that strain HTF-F produces an extracellular polymeric matrix (EPM) that might provide an advantage to colonization *in vivo* through intercellular aggregation and biofilm formation. As bacterial EPMs are common components of biofilm matrices and have been previously shown to have immunomodulatory effects [8–10], we tested the EPM of strain HTF-F for its potential anti-inflammatory effects in the DSS colitis model. Additionally, the immunomodulatory effects of the EPM were investigated using immature dendritic cells derived *ex vivo* from human monocytes and mouse bone marrow cells.

## Materials and Methods

### Animals

BALB/c mice were reared in conventional conditions. Two-month-old females were used for these studies and their body weights were measured before and after each experiment. Animal experiments were approved by the Ethical Committee of the Institute of Microbiology, Academy of Sciences of the Czech Republic.

### Bacterial strains and culturing conditions

*F*. *prausnitzii* strain HTF-F and A2-165 have been described elsewhere [11–13] and were maintained at 37°C on yeast extract, casitone, fatty acid and glucose medium (YCFAG, described in [13]) under anaerobic conditions. For EPM production, *F*. *prausnitzii* strains were cultured in YCAG broth, which have the same composition as YCFAG medium but, except acetate, all short chain fatty acids were omitted. *L*. *plantarum*WCFS1 was cultured overnight until stationary phase in MRS broth (Merck, Darmstadt, Germany) at 37°C. Bacteria were harvested by centrifugation at 4°C, 3300 g for 15 min, washed in PBS, resuspended in PBS containing 20% glycerol and stored at -80°C prior to use. For the BMDCs assays, *L*. *plantarum*WCFS1 grown in MRS at 37°C overnight were inactivated with 1% formaldehyde-PBS as described previously [14]. Bacteria were quantified by fluorescent *in situ* hybridization (FISH) or phase contrast microscopy. All buffers and media used for the anaerobic bacteria were deoxygenated by flushing with oxygen free nitrogen for 30 minutes.

### Isolation and staining of the *F*. *prausnitzii* extracellular polymeric matrix

The cell bound EPM was extracted as previously described [15]. Briefly, 250 ml of 24 h cultures of *F*. *prausnitzii* strains HTF-F or A2-165 were recovered by centrifugation at 18400 g for 10 min (4°C). The pre-washed cell pellet was suspended in 8 ml of PBS by vortexing for 5 min allowing the cell bound EPM to dissolve. Planktonic cells were subsequently pelleted by centrifugation at 18400 g for 10 min (4°C). The supernatant was then carefully removed and added to 4 volumes of ice cold absolute ethanol to precipitate the EPM. After centrifugation at 3300 g for 30 min, the EPM precipitated pellet was washed with 70% ethanol, then lyophilized and stored at -20°C. For further experiments, lyophilized EPM fractions were dissolved in PBS at the desired concentrations. The EPM yield was 1.2 mg/ml from approximately 2.5 x 10^11^ bacteria. The EPM was shown to be free of bacterial contamination by visual inspection after Gram staining. TLR assays (described in S1 Materials and Methods) showed that the EPM was free of contaminating microbe-associated molecular patterns (MAMPs).

### Human DCs assays

This study was approved by Wageningen University Ethical Committee and was performed according to the principles of the Declaration of Helsinki. Buffy coats from blood donors were obtained from the Sanquin Blood bank in Nijmegen (The Netherlands). A written informed consent was obtained before sample collection. The expression of surface markers and the cytokines secreted in the supernatant were measured after incubation of hDCs with *F*. *prausnitzii* A2-165 (hDCs from 3 donors), HTF-F (3 donors), *L*. *plantarum* (5 donors), EPM (3 donors) or *L*. *plantarum* together with EPM (5 donors). Bacteria were used at a bacterium: DC ratio of 10:1 and the EPM at 14.4 μg/ml (1.2% v/v). Mononuclear cells were isolated from buffy coats of healthy donors using Ficoll Paque Plus density gradient (GE Healthcare, Diegem, Belgium) according to the manufacturer’s protocol. After centrifugation, mononuclear cells were collected and monocytes were isolated by positive selection of CD14^+^ cells using CD14-specific antibody coated magnetic microbeads (Miltentyi Biotec, Leiden, The Netherlands). CD14^+^ cells were cultured for 6 days in complete medium in the presence of IL-4 and granulocyte-macrophage colony-stimulating factor (GMCFS, R&D Systems, Minneapolis, MN) to differentiate into immature monocyte-derived DCs. At day 6, cells were seeded at 10^6^ cells/ well in 24-well plates and were treated with *L*. *plantarum* (bacterium: DC, 10:1) in the presence or absence of the EPM isolated from *F*. *prausnitzii* (1,2% v/v) or left untreated. After 48 hours of co-incubation, the supernatant was collected for cytokine measurements. During the culture period and the stimulation, DCs were cultured in RPMI 1640 culture medium (Invitrogen) supplemented with 10% FCS, 100 U/ml penicillin and 100 μg/ml streptomycin (Sigma, St. Louis, MO) and no bacterial growth was observed. On day 6 and 8, the activation and maturation status of the CD14^+^ cells were assessed by measuring CD83 and CD86 surface expression and the cell viability was measured using Annexin V and propidium iodide (PI). Cells were stained with fluorescence conjugated monoclonal antibodies specific for CD83, CD86, their isotype-matched controls and with annexin V and PI (BD Biosciences, Breda, The Netherlands) and analysed on a flow cytometer (FACS Canto II, BD). CD86 and CD83 were expressed at low levels on immature or untreated DCs and were highly expressed after stimulation. On days 6 and 8 the viability of the cells was between 60–80% (not shown).

### RNA isolation and real-time qPCR

Total RNA was isolated from hDCs using the RNAeasy Mini Kit (Qiagen) following the manufacturer instructions. cDNA synthesis was performed using 500 ng of isolated total RNA and Q-script (Quanta bioscience) according to the manufacturer instructions. cDNA was diluted in nuclease-free water to a final volume of 100 μl and stored at -20°C until further use. Primers for IL-10 (forward 5’-GTGATGCCCCAAGCTGAGA-3’, reverse 5’-CACGGCCTTGCTCTTGTTTT-3’), IL-12p40 (forward 5’-CTCTGGCAAAACCCTGACC-3’, reverse 5’-GCTTAGAACCTCGCCTCCTT-3’), IL-1β (forward 5’-GTGGCAATGAGGATGACTTGTTC-3’, reverse 5’-TAGTGGTGGTCGGAGATTCGTA-3’), TNF-α (forward 5’- CTGCTGCACTTTGGAGTGAT-3’, reverse 5’-AGATGATCTGACTGCCTGGG-3’), and the reference genes GAPDH (forward 5’-CTGCACCACCAACTGCTTAG-3’, reverse 5’-GTCTTCTGGGTGGCAGTGAT-3’) and β-actin (forward 5’-TTGCGTTACACCCTTTCTTG-3’, reverse 5’-CACCTTCACCGTTCCAGTTT-3’) were designed using PRIMER3 software [16]. Quantitative RT-PCR (qPCR) was performed using the GoTaq qPCR mastermix (Promega), briefly, 5 μl cDNA (20x dilution), forward and reverse primers (300 nM each) were added to 7 μl qPCR mastermix and demineralised water was added to a final volume of 14 μl. The qPCR reaction was carried out on a Rotorgene 6000 real-time cycler (Qiagen). Raw data were analysed using the comparative quantitation method of the Rotor-gene Analysis Software V5.0 and relative gene expression levels were determined as ratio of target gene vs. reference gene and were calculated according to the ΔCt method [17] using the following equation:

$$Ratio\;=\;\frac{{\left(E_{target}\right)}^{{Ct}_{target}(control-sample)}}{({E_{reference})}^{{Ct}_{reference}(control-sample)}}$$

Where E is the amplification efficiency and Ct is the number of PCR cycles needed for the signal to exceed a predetermined threshold value. Dual internal reference genes (GAPDH and β-actin) were incorporated in all qPCR experiments and results were similar following standardization to either gene. For each sample a controls that was not treated with reverse transcriptase was included and no amplification above background levels was observed. Non-template controls were included for each gene in each run and no amplification above background levels was observed. Specificity of the amplification was ensured by checking the melting temperature and profile of each melting curve. The product of each template was checked at least once by sequencing.

### Mouse BMDCs assays

Mouse BMDCs from BALB/c mice were prepared as previously described [18]. Briefly, bone marrow cells isolated from femurs and tibias were seeded at 2 x 10^5^ cells/ml in bacteriological Petri dishes in RPMI 1640 medium containing 10% fetal bovine serum, 150 μg/ml gentamycin and 20 ng/ml mouse rGMCSF (Sigma-Aldrich, Munich, Germany). Fresh medium was added at day 3 and 6 and BMDC were used on day 8 of culture. Where indicated BMDCs (10^6^ cells/ml) were incubated with anti-TLR2 antibody (InvivoGen) or control isotype antibody IgG2a (eBioscience, San Diego, CA) at concentration 10 μg/ml for 1 hour 37°C prior to stimulation with *L*. *plantarum*, EPM or *L*. *plantarum* together with EPM for 20 h. *L*. *plantarum* was used at a bacterium: DC ratio of 10:1, EPM at 1.2% v/v. Culture supernatants of stimulated BMDC were stored at -20°C until use.

### Cytokine analysis

Cytokines concentrations in the hDC culture supernatants were determined using Cytokine bead arrays (BD) and a flow cytometer (FACS Canto II, BD). In hDCs studies, the limits of detection were the following: for IL-1β 7.2 pg/ml, for IL-10 3.3 pg/ml, for TNF 3.7 pg/ml and for IL-12p70 1.9 pg/ml. Mouse IL-10 was measured in culture supernatants by enzyme-linked immunosorbent assay (ELISA) using Ready-Set-Go! Kit (eBioscience) according to manufacturer’s instructions. Levels of mouse IL-12p70 were measured with matched antibody pairs (BD).

### Intrarectal administration of bacteria or EPM and induction of acute ulcerative colitis

The experimental groups of 10 mice and their respective treatments are shown in Table 1. Mice from groups 2, 3, 4 and 5 received 2.5% DSS (molecular weight 40 kDa; ICN Biomedicals, Ohio, USA) in the drinking water ad libitum for one week. Mice from the untreated control group 1 received only drinking water. Mice from groups 3 and 4 received intrarectally (via tubing) daily doses of 2 to 3 x 10^9^ CFU of *F*. *prausnitzii* HTF-F and A2-165, respectively in 100 μl PBS for ten days prior the DSS exposure and during the eight days of DSS treatment. Mice from group 5 received intrarectally daily doses of 50 μg the EPM in 100 μl PBS for ten days prior the DSS exposure and during the eight days of DSS treatment. Mice from the colitis control group received intrarectally 100 μl PBS. The following clinical symptoms were measured or assessed: firmness of faeces, rectal prolapses, rectal bleeding and colon length after the sacrifice. The colon descendens was taken for myeloperoxidase assay, isolation of mRNA, histological assessment and for intestinal fragment cultivation.

**Table 1:** DSS-induced colitis experimental groups.

Group	Intrarectal treatment	DSS
**1**	PBS	-
**2**	PBS	+
**3**	*F*. *prausnitzii* HTF-F	+
**4**	*F*. *prausnitzii* A2-165	+
**5**	EPM	+

### Disease activity index

Disease activity index (DAI), measured according to Cooper et al. [19], is a combined score of weight loss, stool consistency and bleeding divided by 3. Acute clinical symptoms are diarrhoea and/or grossly bloody stools. The scores are explained in Table 2.

**Table 2:** Scoring of DAI (modified according to Cooper et al. 1993).

Score	Weight loss	Stool consistency*	Occult/gross bleeding
**0**	None	Normal	Normal
**1**	1–5%	Normal	Normal
**2**	5–10%	Loose	Hemacult +
**3**	1–20%	Loose	Blood in colon, starting bleeding from anus
**4**	> 20%	Diarrhoea	Gross bleeding

* Normal stools, well-formed pellets; loose stools, pasty and semi-formed stools which do not stick to the anus; diarrhoea, liquid stools that stick to the anus.

### Histological evaluation of colon damage

Colon tissue was fixed in Carnoy’s fluid for 30 min, transferred into 96% ethanol and embedded in paraffin. Five-mm paraffin-embedded sections were cut and stained with haematoxylin and eosin (H&E) and Alcian Blue and poststained with NuclearFastRed (Vector, Burlingame, CA) for mucin production. Samples were examined using an Olympus BX 40 microscope equipped with an Olympus Camedia DP 70 digital camera, and the images were analysed using Olympus DP-Soft. The degree of damage to the surface epithelium, crypt distortion and mucin production in individual colon segments were evaluated according to Cooper et al. [19].

### Measurement of cytokine production in colonic fragments

Pre-weighted colonic fragments were cultured in RPMI medium enriched with 10% bovine serum albumin in 5% CO2 and 95% air at 37°C, in 24-well flat-bottomed plates (Nunc) for 48h. Culture supernatants were harvested for analysis of their cytokine content by the MILLIPLEX MAP Mouse Cytokine/Chemokine Panel (Millipore, Schwalbach, Germany) according to manufacturer’s instructions and analysed with the Bio-Plex System (Bio-Rad, Paris, France).

### Statistics

Data were analysed using the GraphPad PRISM (Graphpad software, San Diego, CA) by one way Anova followed by Dunnett’s multiple comparison test for the *in vitro* assays or by the Tukey’s test for the *in vivo* assays. Gene expression levels in time and mice body weight changes in time were compared using two way Anova followed by Bonferroni’s test.

## Results

### Phenotypic characteristics of *F*. *prausnitzii* strain HTF-F and purification of the extracellular polymeric matrix

**Fig 1:**
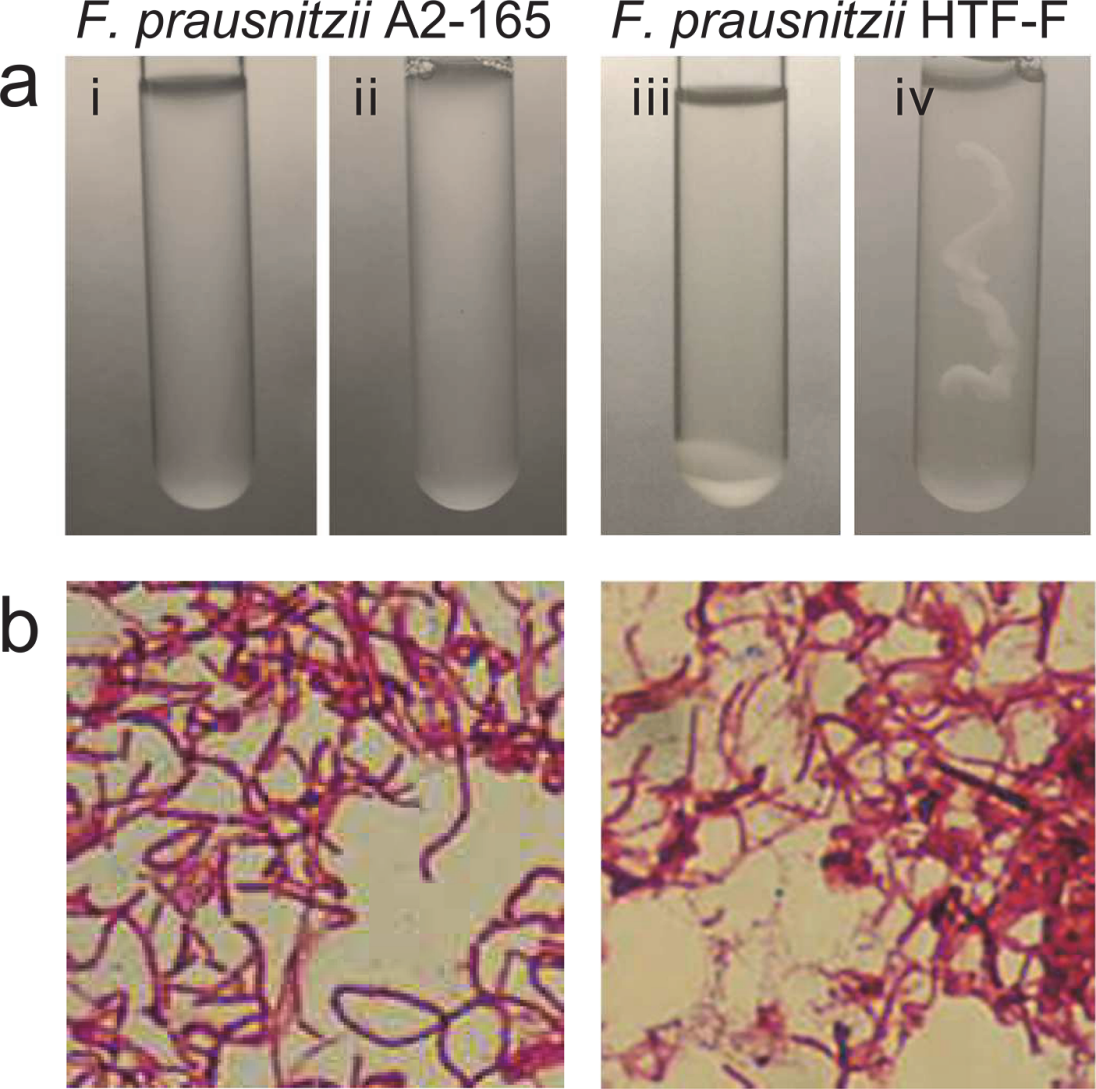
Phenotypic characteristics of *F*. *prausnitzii* strain HTF-F and A2-165. a) Growth and biofilm formation of *F*. *prausnitzii* strains HTF-F and A2-165 in YCFAG medium under anaerobic conditions. i and ii) *F*. *prausnitzii* A2-165 before and after shaking respectively; iii and iv) *F*. *prausnitzii* HTF-F before and after shaking respectively. b) Gram staining of *F*. *prausnitzii* A2-165 (left panel) and HTF-F (right panel).

**Fig 2:**
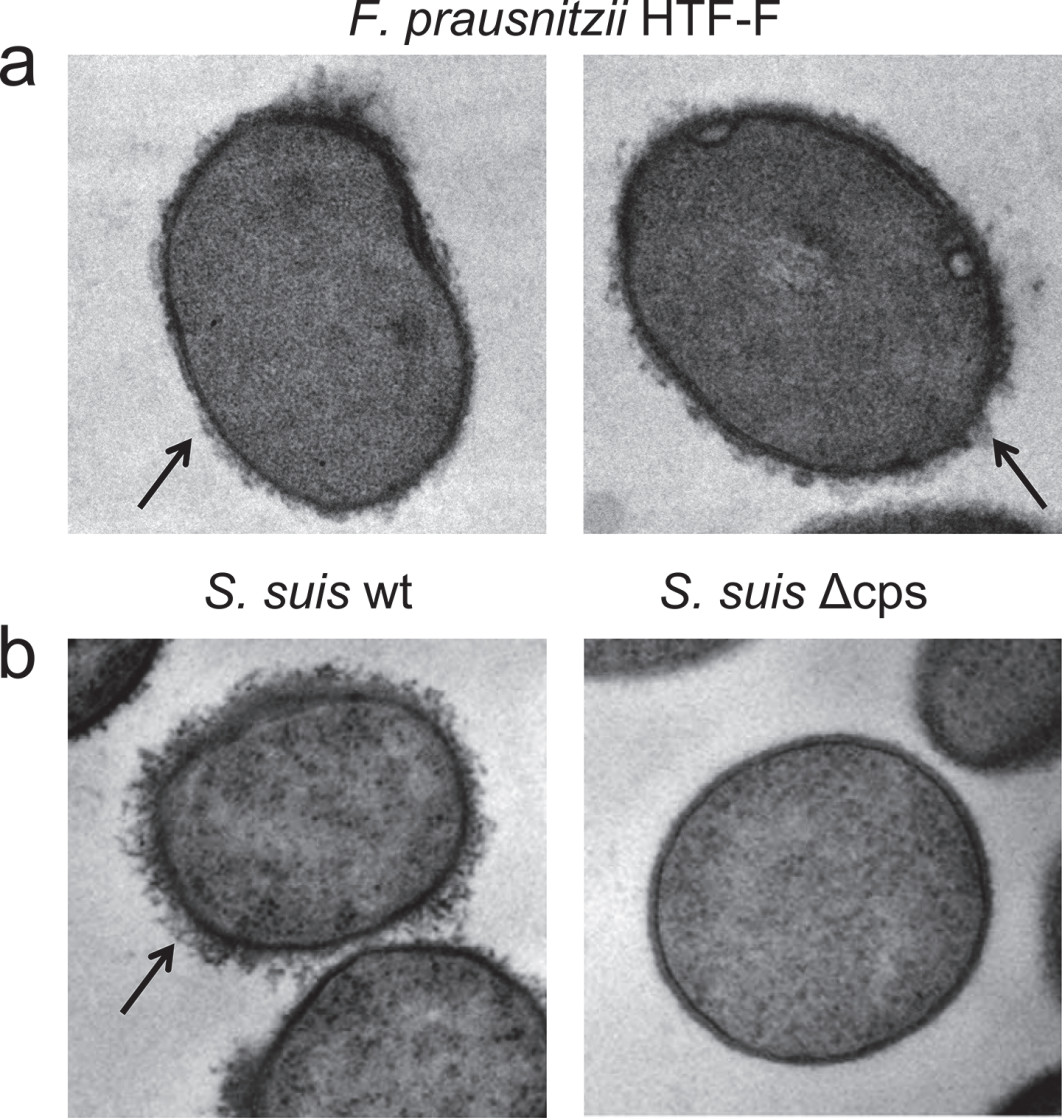
Detection of *F*. *prausnitzii* HTF-F EPM by transmission electron microscopy. *F*. *prausnitzii* HTF-F (a) possess a diffuse and irregular surface layer (arrow) which is thinner but similar to the capsule polysaccharide (CPS) of *S*. *suis* wild type strain (arrow, *S*. *suis* wt, left panel b) and absent in *S*. *suis* CPS deletion mutant (*S*. *suis* Δcps, right panel b).

**Fig 3:**
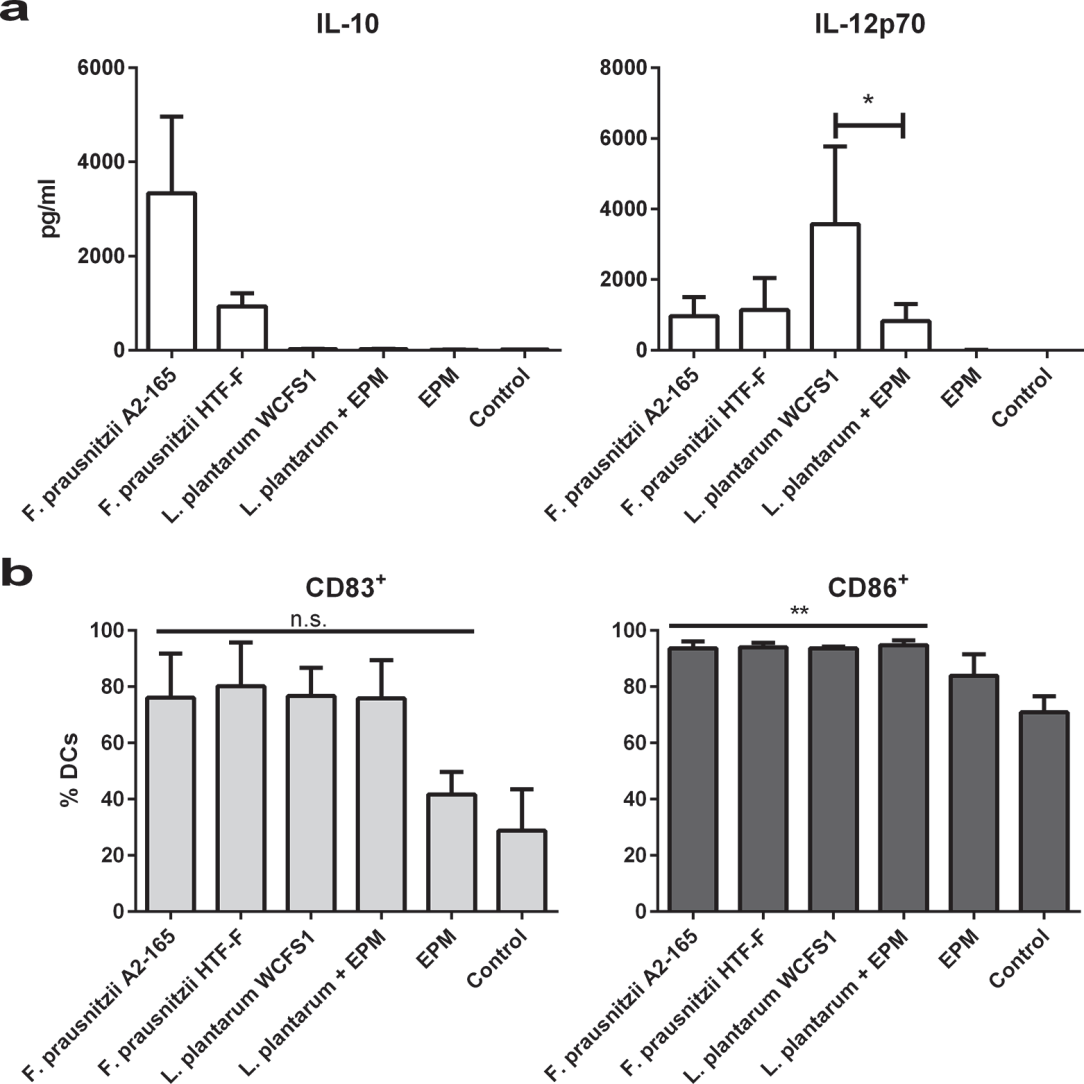
Cytokine secretion and surface marker expression in hDCs. Cytokine secretion and surface marker expression in hDCs after 48 h of incubation with *F*. *prausnitzii* A2-165 (3 donors), *F*. *prausnitzii* HTF-F (3 donors), *L*. *plantarum* (5 donors), *L*. *plantarum* + EPM (5 donors), EPM (3 donors) or left unstimulated (5 donors). a) IL-10 and IL-12p70 were measured in the supernatant of hDCs. Error bars represent SEM, * indicates p<0.05 compared with *L*. *plantarum* treated samples. b) Percentage of CD83^+^ (left panel) and CD86^+^ (right panel) hDCs. Error bars represent SEM, ** indicates p<0.01, n.s. indicates non-significant compared with the control.

*F*. *prausnitzii* HTF-F and A2-165 form colonies with mucoid appearance on solid agar, however, only strain HTF-F forms a mucoid biofilm in liquid culture (Fig 1A). This phenotype is commonly associated with the production of extracellular polysaccharides and intercellular aggregating proteins [20]. The EPM of strain HTF-F is revealed by Gram staining (Fig 1B) and is observed in transmission electron micrographs as a diffuse and irregular surface layer (Fig 2A, arrow) resembling the capsule polysaccharide (CPS) of *Streptococcus suis* (Fig 2B [21]). The cell bound the EPM produced by strain HTF-F was isolated, concentrated and filtered to remove possible bacterial contaminants. Luciferase-based TLR signalling assays for human TLR2, TLR2/6, TLR4 and TLR5 indicated that MAMPs were not present in amounts that would influence the activation of immune cells *in vitro* (S1 Fig). This was confirmed by the fact that the EPM did not induce cytokine secretion or expression of maturation and activation markers after incubation with hDCs (Fig 3A and 3B).

### The EPM of *F*. *prausnitzii* HTF-F decreases transcription and production of pro-inflammatory IL-12p70 in *L*. *plantarum*-activated hDCs

The immunomodulatory properties of *F*. *prausnitzii* A2-165, HTF-F and the EPM were tested *in vitro* using human monocyte-derived DCs (hDCs). DCs were chosen because they are one of the most important antigen presenting cells with the capacity to prime naive T cells at mucosal sites and to drive the immune response. Incubation of hDCs with *F*. *prausnitzii* A2-165 and HTF-F induced large amounts of IL-10 and small amounts of IL-12p70 compared with incubation with *L*. *plantarum* (Fig 3A) or other lactobacilli (not shown). The amount of IL-10 produced after incubation of hDCs with strain HTF-F was lower than with strain A2-165 (Fig 3A). In these experiments, live bacteria were used but they were rapidly killed by the presence of antibiotics in the culture medium, this is necessary to prevent bacterial overgrowth. Contact of bacteria with DCs and phagocytosis occurs quite rapidly (30 min to 1h) so bacteria are not very different to their live counterparts *in vivo*.

**Fig 4:**
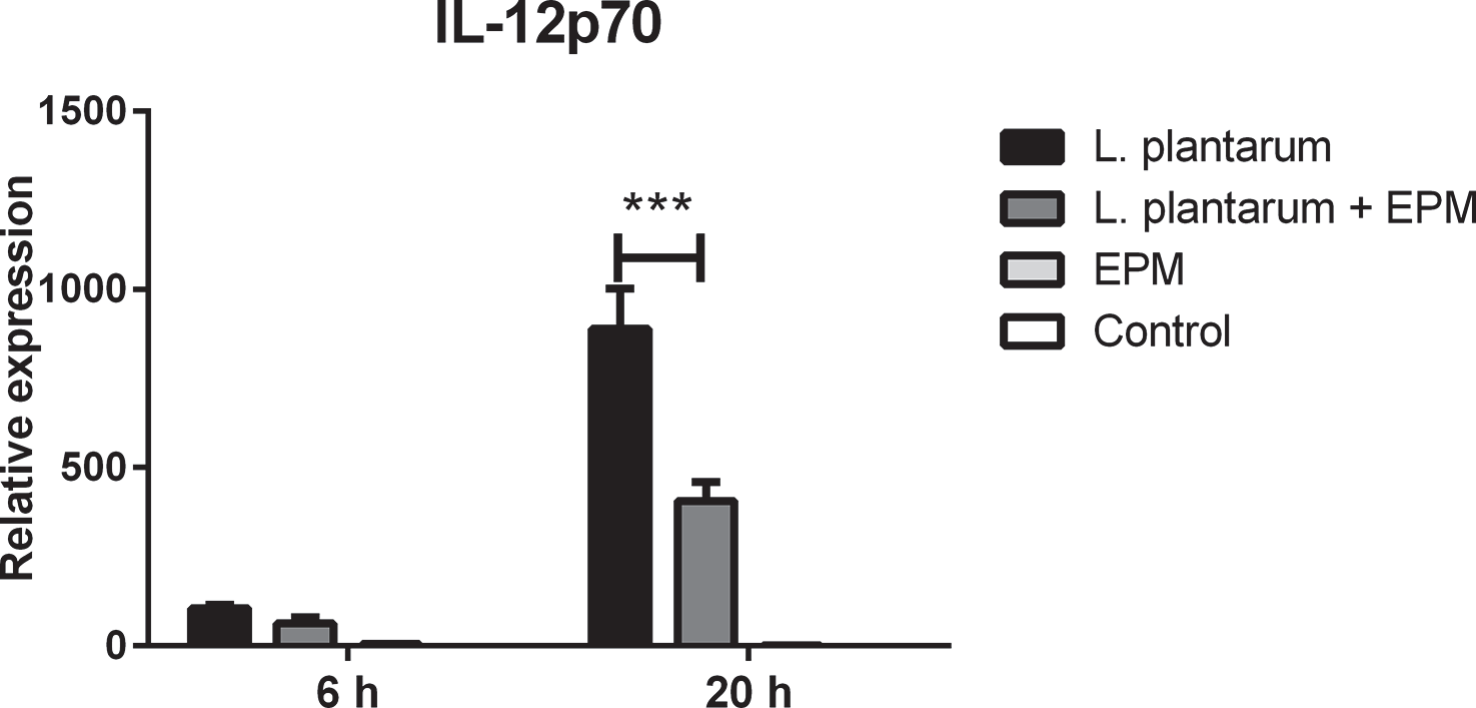
Relative gene expression levels in hDCs determined by quantitative RT-PCR. RNA was extracted from hDCs after 6 and 20 h of incubation with *L*. *plantarum* (in black), *L*. *plantarum* + EPM (in dark grey), EPM (in clear grey) or from unstimulated cells (in white) and the expression levels of IL-12p70 gene was calculated relative to the expression levels of the housekeeping gene GAPDH. Error bars represent SEM, n = 3, *** indicates p<0.001 compared with *L*. *plantarum* treated samples.

The EPM alone had no effect on the expression of activation or maturation markers and cytokine expression compared to untreated hDCs (Fig 3B) confirming the lack of TLR signalling activity (S1 Fig). Therefore, the EPM was combined with *L*. *plantarum* in hDC cultures to investigate whether it would modulate cytokine production. In combination with *L*. *plantarum*, the EPM reduced the secretion of pro-inflammatory IL-12p70 but had no effect on production of IL-10, which appeared not to be induced by *L*. *plantarum* in hDCs (Fig 3A). Cytokines IL-1β and TNF-α were elicited by incubation of hDCs with *L*. *plantarum* but were unaffected by addition of EPM (not shown). The effect of EPM on IL-12p70 production by *L*. *plantarum*-stimulated hDCs was not due to altered maturation and activation as evidenced by the measurement of the co-stimulatory molecules CD83 and CD86 (Fig 3B). To investigate whether the reduced secretion of IL-12p70 was due to transcriptional regulation, quantitative RT-PCR was performed on IL-10, IL-12, IL-1β and TNF-α mRNA extracted from hDCs at 6 and 20 h after incubation with *L*. *plantarum*, *L*. *plantarum* combined with the EPM, EPM alone or unstimulated hDCs. The addition of the EPM had no significant effect on transcription of IL-1β, TNF-α or IL-10 in hDCs stimulated with *L*. *plantarum* (not shown) but significantly decreased the transcript levels of IL-12 at 20h by about 2 fold (Fig 4).

### The immunomodulatory effects of the EPM are TLR2 dependent

**Fig 5:**
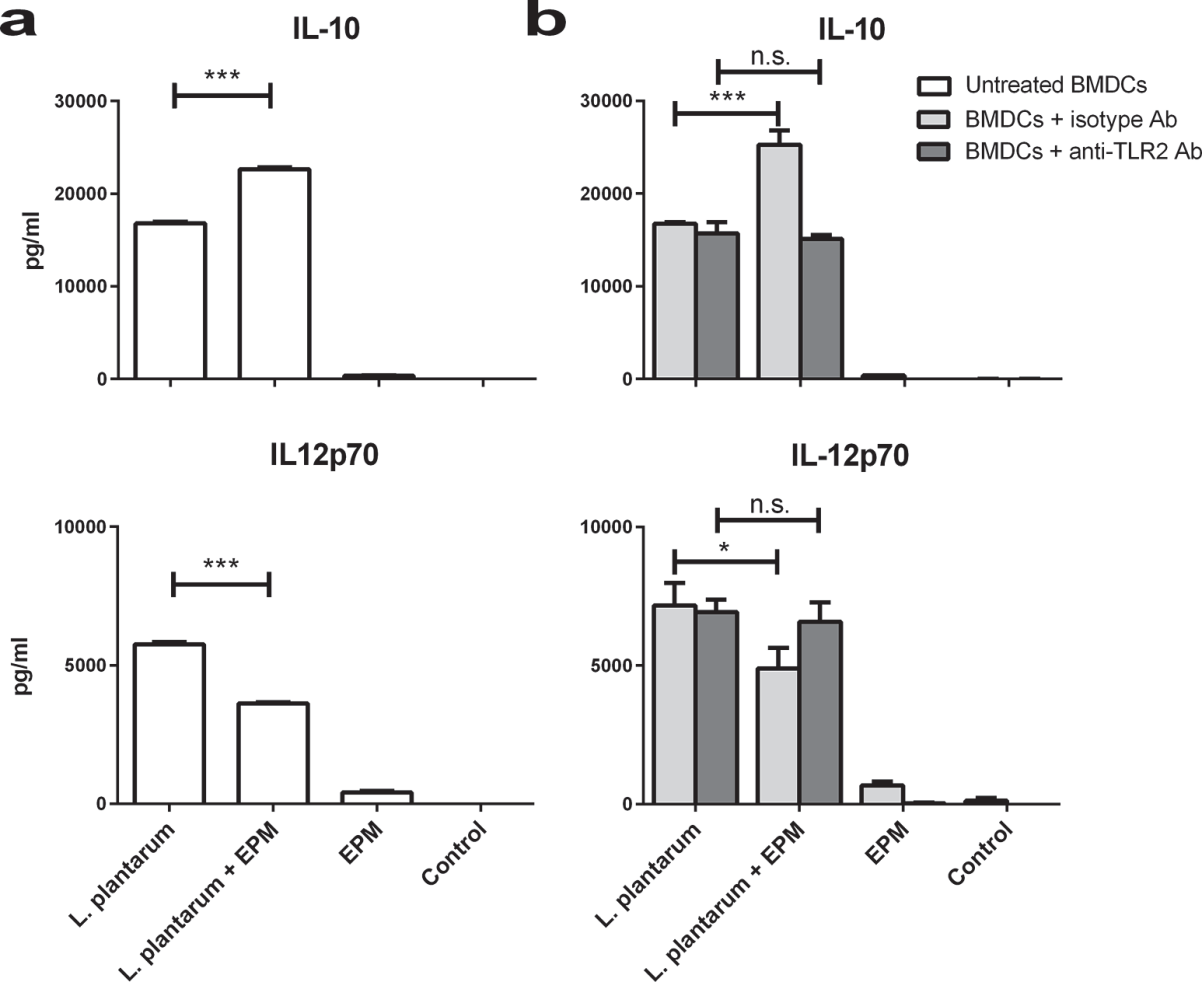
Cytokine secretion in mouse BMDCs with and without anti-TLR2 blocking antibody. a) IL-10 and IL-12p70 were measured in BMDC supernatants after incubation with *L*. *plantarum*, *L*. *plantarum* + EPM, EPM and unstimulated DCs. b) IL-10 and IL-12p70 were measured after incubation of BMDCs with the same samples as in panel (a) except that anti-TLR2 blocking antibody (anti-TLR2 Ab, dark grey bars) or an isotype control (isotype Ab, clear grey bars) were included during the incubation period. Error bars represent SEM (n = 3), *** indicates p<0.001, * indicates p<0.01, n.s. non-significant compared to *L*. *plantarum* treated samples.

To investigate the anti-inflammatory effects of the EPM on IL-12p70 production by hDCs, we performed an experiment using mouse bone marrow-derived DCs (BMDCs) stimulated with *L*. *plantarum* with or without the EPM. As found using hDCs, the presence of the EPM reduced the secretion of pro-inflammatory IL-12p70 by mouse BMDCs stimulated with *L*. *plantarum*. Strikingly, the EPM also increased the production of IL-10 in BMDCs stimulated with *L*. *plantarum* which was already induced at much higher levels than in hDCs stimulated with *L*. *plantarum*. These effects were not due to the induction of cytokine secretion by the EPM alone (Fig 5A). Additionally, we tested whether the immunomodulatory effects of the EPM were dependent on TLR2 signalling by including a TLR2 blocking antibody or an irrelevant antibody of the same isotype in the assays. The effects of the EPM on IL-12p70 and IL-10 production by *L*. *plantarum* stimulated BMDCs were inhibited in the presence of TLR2 blocking antibody but not in the presence of the isotype antibody. As the EPM did not induce TLR2 signalling, the mechanism leading to reduced transcription of IL-12p70 and increased production of IL-10 was presumably dependent on TLR2 signalling by *L*. *plantarum* (Fig 5B). The fact that *L*. *plantarum* could still activate immune cells and induce cytokines in the presence of a TLR2 blocking antibody could be explained by intracellular pattern recognition receptors including NLRs and other TLRs in the phagosomal compartment.

### *F*. *prausnitzii* and the EPM of strain HTF-F attenuate clinical symptoms in DSS-colitis

**Fig 6:**
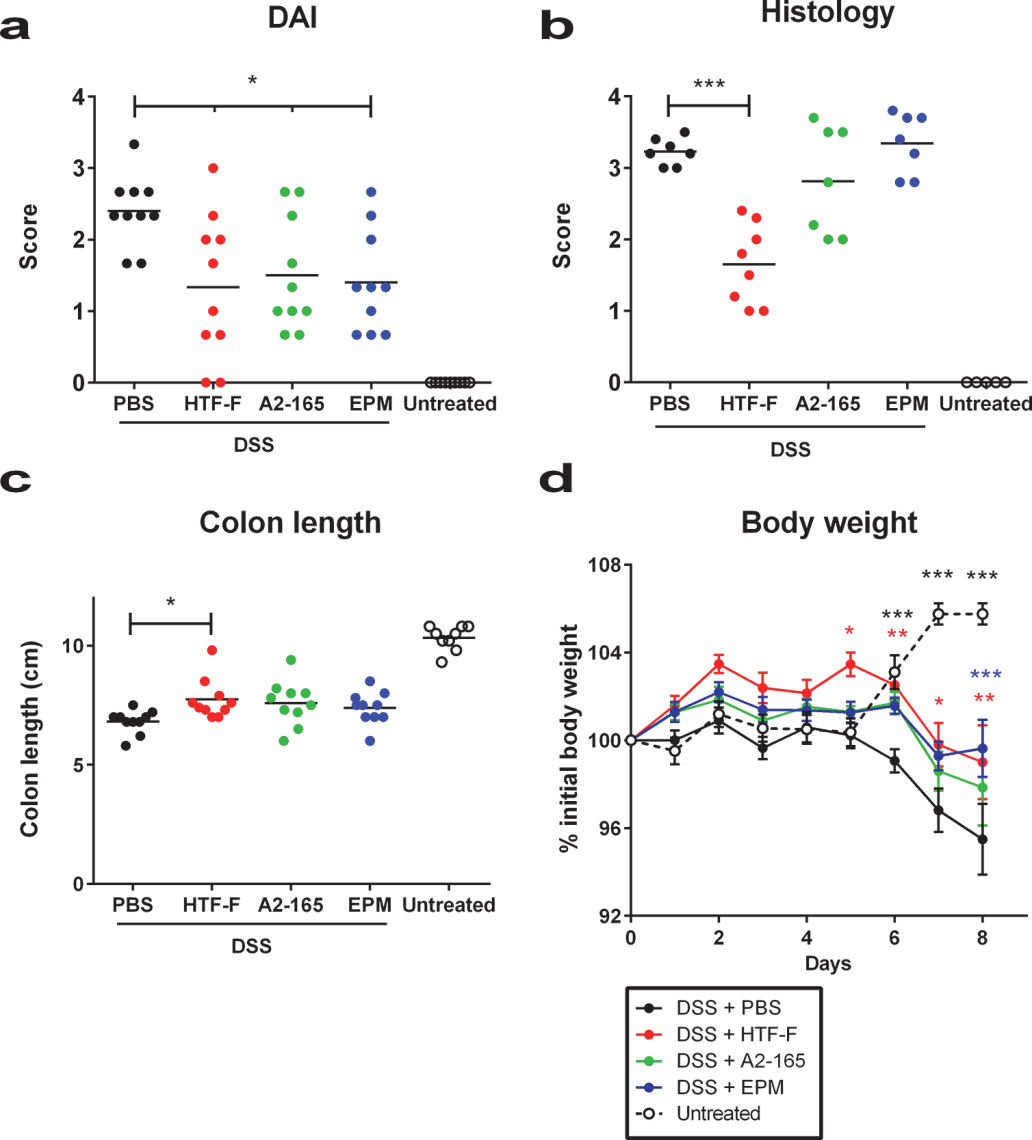
Clinical symptoms of DSS-colitis. Disease activity index (DAI), colon histological damage score and clinical evaluation of DSS treated mice. Mice were left untreated (in white) or treated with DSS during 8 days and administered intrarectally with PBS (in black), EPM (in blue) or *F*. *prausnitzii* strains HTF-F (in red) or strain A2-165 (in green). DAI, histological score and colon length (a, b and c respectively) were evaluated at the end of the experiment. Mice body weight (d) was measured throughout the experiment, body weight values are expressed as percentage of the initial value measured at day 0 before DSS administration. Error bars represent SEM, n = 10, * indicates p<0.05, ** p<0.01, *** p<0.001 compared with the control colitis mice that received DSS + PBS.

**Fig 7:**
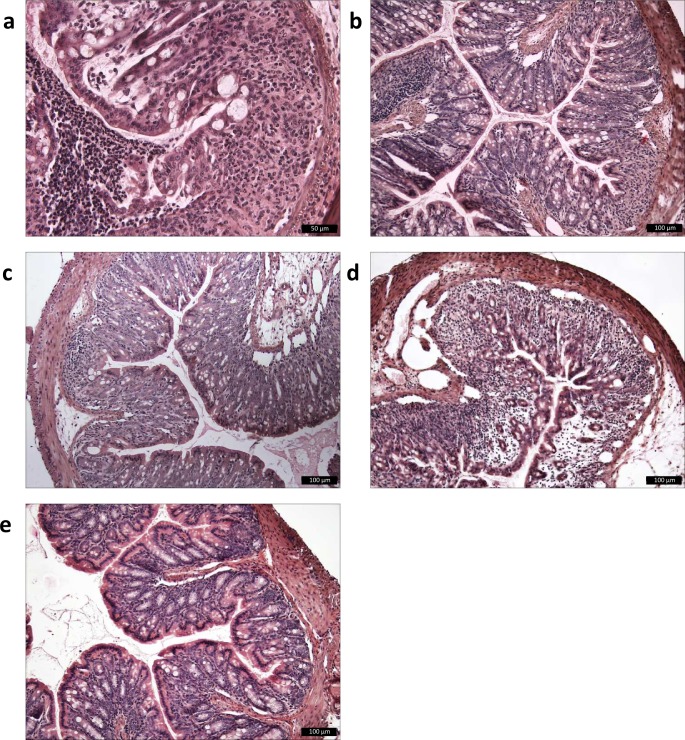
Histological colonic sections of DSS-treated mice. Histological cross-sectional views of colon descendens of untreated or DSS-treated mice: a) colitis control, PBS-DSS-treated mice (damage grade 3–3.5) b) HTF-F-DSS-treated mice (damage grade 1–2.4); c) A2-165-DSS-treated mice, (damage grade 2–3.7); d) EPM-DSS-treated mice (damage grade 2.8–3.8); e) untreated mice (damage grade 0).

The potential protective effects of *F*. *prausnitzii* strains A2-165, HTF-F and EPM were assessed in mice using the DSS-induced colitis model. The bacteria or the EPM were administered to mice intrarectally ten days prior to DSS exposure and continuously administered daily over a period of eight days in which DSS was given in the drinking water to induce colitis. The severity of colitis was evaluated for individual mice in each group by measuring disease activity index (DAI), histological damage score of the colon, body weight and colon length. *F*. *prausnitzii* A2-165, HTF-F and EPM administration significantly decreased the DAI compared to colitis control mice which received PBS intrarectally and DSS in the drinking water, although the score was higher than in untreated mice (Fig 6A). The histological colon damage score was grade 0 in untreated mice (Figs 6B and 7A). *F*. *prausnitzii* HTF-F administration significantly decreased colon damage score compared to the colitis control mice (grade 1.65 and 3.2 respectively, Figs 6B, 7C and 7B), while *F*. *prausnitzii* A2-165 and EPM administration did not significantly affect the colon damage score compared to colitis control mice (grade 2.8 and 3.3 respectively, Figs 6B, 7D and 7E).

The colon length was reduced in all DSS treated groups compared to untreated mice but the *F*. *prausnitzii* HTF-F treated group had significantly longer colons compared with colitis control mice (Fig 6C) indicating a reduced severity of colitis. The body weight of mice was measured throughout the period of DSS treatment and compared to the weight before treatment. In untreated mice, the body weight increased by approximately 5% from day 5 to day 8. However, in colitis control mice, the body weight decreased by approximately 5% from day 5 to day 8. In mice administered *F*. *prausnitzii* HTF-F, A2-165 or EPM, the decrease in body weight was delayed by one day compared to the colitis control mice and for mice treated with *F*. *prausnitzii* HTF-F or EPM the final body weight on day 8 was significantly higher than that of the colitis control group (Fig 6D). Taken together these results indicate that *F*. *prausnitzii* strains A2-165, HTF-F as well as the EPM can attenuate the clinical symptoms of DSS-induced colitis.

### Effects of *F*. *prausnitzii* and *F*. *prausnitzii* EPM on Foxp3 expression in mesenteric lymph nodes and spleen of DSS-treated mice

**Fig 8:**
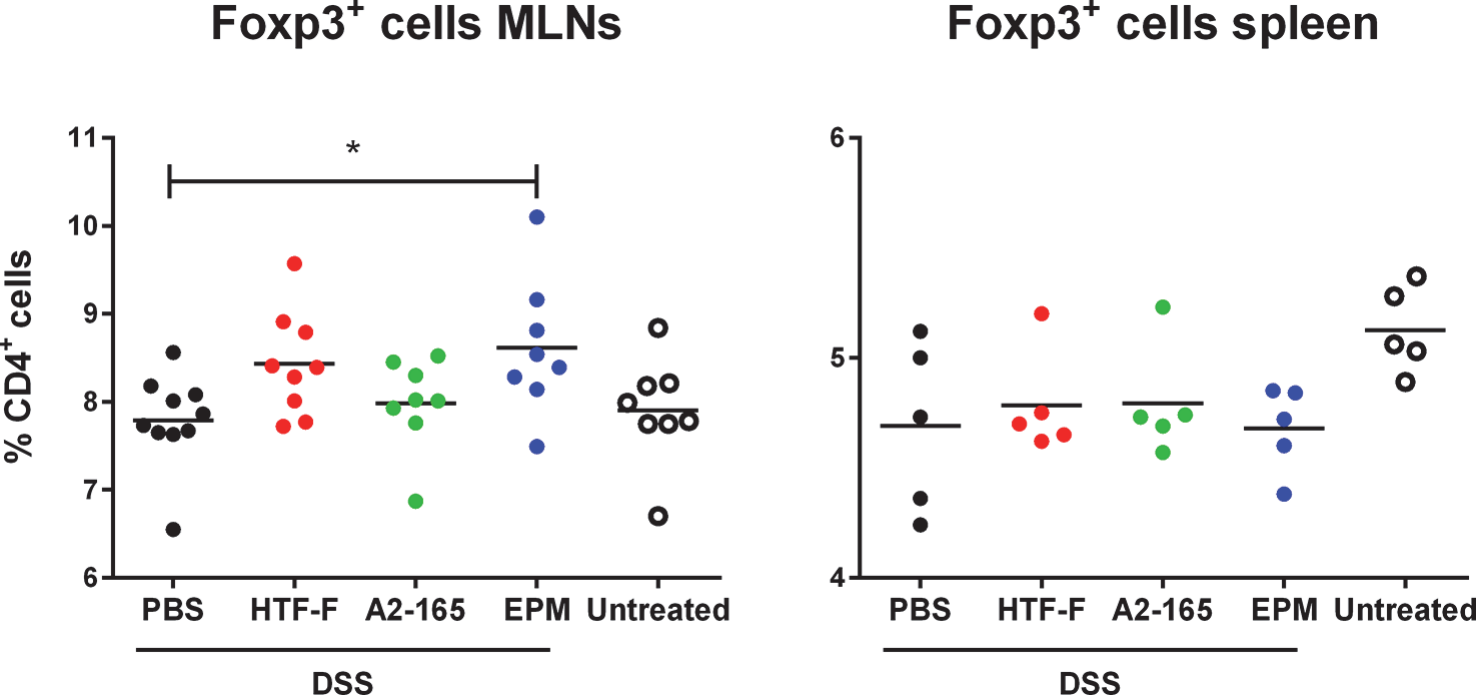
Foxp3 expression in mesenteric lymph nodes and spleen of DSS-treated mice. Percentage of Foxp3^+^ CD4^+^ T cells isolated from mesenteric lymph nodes (MLNs, left panel) and spleens (right panel) of mice untreated (in white) or treated with DSS during 8 days and administered intrarectally with PBS (in black), EPM (in blue) or *F*. *prausnitzii* strains HTF-F or strain A2-165 (HTF-F in red and A2-165 in green, respectively).

To investigate the potential role of Foxp3^+^ Tregs in the attenuation of DSS-induced colitis, we measured the number of CD4^+^ T cells isolated from mesenteric lymph nodes (MLNs) and spleens that express intracellular Foxp3 by fluorescence-activated cell sorting (FACS) analysis. DSS treatment did not significantly affect the levels of Foxp3^+^ CD4^+^ T cells in the MLNs or spleen compared to untreated mice. Administration of *F*. *prausnitzii* also had no effect on Foxp3^+^ CD4^+^ T cells compared to untreated mice. However, administration of EPM induced a small but significant increase in Foxp3^+^ CD4^+^ T cells in the MLNs but not in the spleen (Fig 8).

### *F*. *prausnitzii* HTF-F decreases the secretion of IFN-γ and IL-17 from colonic cultures

**Fig 9:**
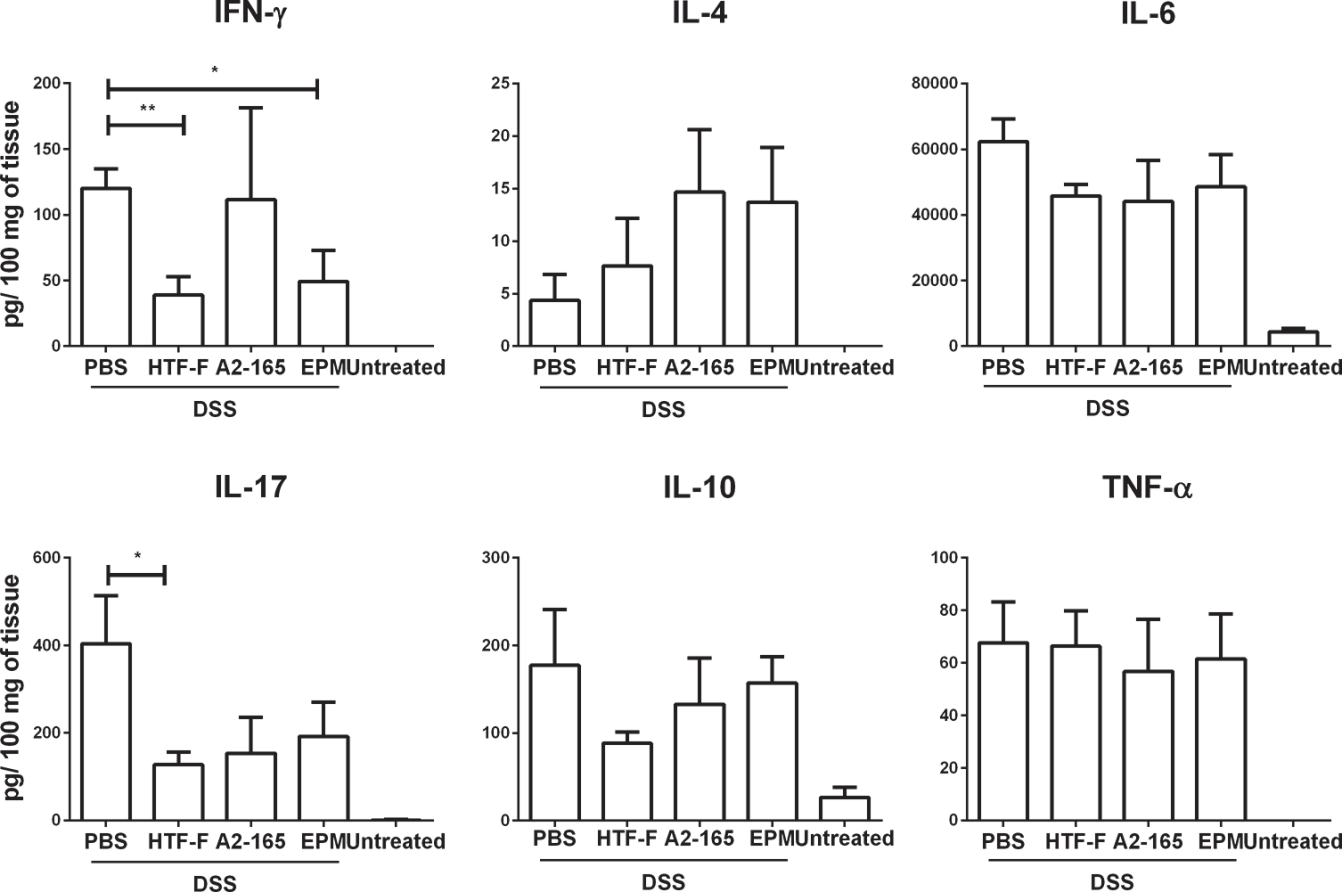
IFN-γ and IL-17 secretion in colon cultures from DSS-treated mice. Mice were left untreated or treated with DSS during 8 days and administered intrarectally with PBS, EPM or *F*. *prausnitzii* strains HTF-F or A2-165. Cytokines were measured in the supernatants of 48h cultures of colonic fragments isolated from mice. Error bars represent SEM, n = 5, * indicates p<0.05, ** p<0.01.

To investigate the effects of the treatments on cytokine secretion in the colon, sections from colon descendens were cultured and the cytokine secretion was measured. DSS treatment significantly increased cytokine secretion from cultures of colon descendens compared to untreated controls. Intrarectal administration of *F*. *prausnitzii* HTF-F and EPM induced a decrease in the IFN-γ secretion and *F*. *prausnitzii* HTF-F induced a decrease in IL-17 secretion compared to PBS administration in the colitis control group. However, *F*. *prausnitzii* or EPM administration did not have effect on IL-4, IL-6, IL-10 or TNF-α secretion (Fig 9).

## Discussion

In this study, we demonstrated that *F*. *prausnitzii* strain A2-165, the biofilm forming strain HTF-F as well as the EPM isolated from strain HTF-F can attenuate the clinical symptoms of DSS-induced colitis. Strain HTF-F was stronger than strain A2-165 or the EPM in suppressing inflammation having significant effects on the colon damage score and colon length compared to the other treatments (Fig 6). The administration of purified EPM decreased the DAI indicating that it contributes to the protective effect of strain HTF-F and may be responsible for the stronger protection seen with strain HTF-F compared to A2-165. However, we cannot rule out other possible strain differences, for example in colonization potential, stress resistance or fitness *in vivo*, which might contribute to the efficacy of immune suppression. Improved survival or colonization of strain HTF-F *in vivo* might impact on the amount of butyrate produced in the colon. Microbially-produced butyrate is considered important for colonic health, and in the prevention of colorectal cancer owing to its use as an energy source for epithelial cells and as a modulator of oxidative stress and inflammation [22]. Moreover, oral administration of sodium butyrate has been recently shown to attenuate inflammation in experimental UC and be effective in therapy of UC patients [23]. Thus, increased butyrate production may have contributed to the anti-inflammatory effects of the *F*. *prausnitzii* strains in experimental colitis model. Previously, *F*. *prausnitzii* A2-165 and its supernatant were shown to attenuate TNBS colitis in mice by daily intragastric administration prior to and during the induction of colitis [7]. In the study of Sokol et al., the colons of mice treated with either *F*. *prausnitzii* A2-165 or its supernatant had a reduced amount of IL-12p70 and an elevated amount of IL-10 compared with the colitis control group. This is compatible with the relatively high amount of IL-10 induced by i*n vitro* culture of *F*. *prausnitzii* A2-165 with hDCs (Fig 3) and PBMCs [7]. IL-10 is fundamental for the maintenance of homeostasis in the intestine [24,25]. It is secreted by DCs as well as Foxp3^+^ and Foxp3^-^ T cells in the lamina propria. Secretion of IL-10 by DCs is important for the maintenance of functional Foxp3^+^ Tregs during intestinal inflammation [26]. IL-10 also inhibits the production of pro-inflammatory cytokines such as IFN-γ, TNF-α, IL-6 and IL-12. Moreover, IL-10 was shown to play a role in controlling pro-inflammatory responses to translocated microbes by abrogating IL-23 production [27]. Nevertheless, differences in IL-10 produced *in vitro* by DCs cultured with *F*. *prausnitzii* A2-165 and strain HTF-F cannot explain the better protection seen with HTF-F as it induced less IL-10 than A2-165. *In vitro* assays indicated that the anti-inflammatory mechanism of the EPM was not due to contamination with MAMPs or activation of DCs. However, when the EPM was added together with *L*. *plantarum* as an inflammatory stimulus to hDCs, it decreased the production of IL-12p70 compared to *L*. *plantarum* alone and had no effect on IL-10, IL-1β and TNF-α production (Fig 3). A similar effect of the EPM was seen on IL-12p70 production by mouse BMDCs but, in addition, IL-10 was significantly increased (Fig 5). The effect of EPM on IL-12p70 production occurred at the transcriptional level (Fig 4) suggesting the involvement of the EPM in cell signalling. This mechanism was dependent on TLR2 signalling although the EPM itself did not induce TLR2 signalling in reporter assays, or activate hDCs or mouse BMDCs as in the case of synthetic TLR2 agonists (Fig 5). *L*. *plantarum* alone still induced cytokines in the presence of the TLR2 blocking antibody, this is most likely due to intracellular pattern recognition signalling after phagocytosis. The mechanism of action of the EPM may involve interaction of carbohydrate structures of the EPM with C-type lectin receptors, some of which are known to modulate cytokine production in response to TLR agonists. In the DSS colitis model, administration of EPM but not *F*. *prausnitzii* increased the number of Foxp3^+^ T cells in the MLNs. Similarly, the extracellular polysaccharide A of *Bacteroides fragilis* [9], has also been shown to expand the number of mucosal Foxp3^+^ T cells and confers a TLR2-dependent protection in mouse colitis models [8,9]. In summary, we demonstrated the anti-inflammatory effect of two different *F*. *prausnitzii* strains in the mouse DSS colitis model. Furthermore, we showed that the anti-inflammatory effect of the biofilm forming strain *F*. *prausnitzii* HTF-F may in part be due to the immune-regulating properties of the EPM. *In vitro* immune assays suggest that the immunomodulatory effects of the EPM on IL-10 and IL-12 cytokine production in antigen presenting cells are mediated through modulation of TLR2 signalling. However, the precise anti-inflammatory mechanism of EPM awaits identification of the active component. The newly described strain *F*. *prausnitzii* HTF-F and the EPM may have a therapeutic use in IBD.

## Supporting Information

S1 Fig TLR signalling properties of *F*. *prausnitzii* HTF-F EPM. (TIF)

S1 Materials and Methods A description of the method used for the TLR assay. (DOCX)
