# Supplementary material for: Faecalibacterium prausnitzii Strain HTF-F and Its Extracellular Polymeric Matrix Attenuate Clinical Parameters in DSS-Induced Colitis
Source: PLoS One. 2015 Apr 24;10(4):e0123013. doi: 10.1371/journal.pone.0123013 (PMC4409148; doi:10.1371/journal.pone.0123013)
Supplement: S1 Materials and Methods — (DOCX) [file pone.0123013.s002.docx]

**Materials and Methods S1**

**TLR signalling assays**

TLR assays were performed using human embryonic kidney cells (HEK293) stably expressing human TLR2/6, TLR2/1, TLR4 or TLR5 (Invivogen) and transfected with a reporter plasmid (pNiFTY, Invivogen) containing the luciferase gene under the control of the NF-κB promoter. HEK293 cells expressing the different TLRs and pNiFTY were incubated with the EPM (1.2 % v/v), the TLR agonists, Pam2CSK4 (Invivogen) for TLR2/6, Pam3CSK4 (Invivogen) for TLR2/1, flagellin (Invivogen) for TLR5 and LPS for TLR4, or medium alone as a control. After 6 hours of incubation, the medium was replaced with Bright glow (Promega), and the luminescence was measured using a Spectramax M5 (Molecular Devices). As a negative control, HEK293 cells not expressing TLRs but harbouring pNiFTY were tested in the same conditions and did not show any luciferase activity. The limits of sensitivity for the TLR reporter cell lines were determined in independent experiments using a dose range for each purified agonist. The limits of detection for these reporter cell lines were determined as follows: 2 ng/ml for Pam2CSK4 (TLR2), 8 ng/ml for flagellin (TLR5) and 50 pg/ml for LPS (TLR4); MAMPs present at concentrations lower than this would not activate immune cells in our *in vitro* assays and, as expected, the EPM did not activate hDCs or mouse BMDCs (Figure 3 and 5).
